# Comparison of the Equivalence of *Aspergillus* Antigen and PCR Results Between Non‐Directed Bronchial Lavage and Bronchoalveolar Lavage—A Prospective Exploratory Pilot Study in Critically Ill Patients

**DOI:** 10.1111/myc.70029

**Published:** 2025-02-03

**Authors:** Maria Schroeder, Mohamad Abd Raboh, Annika Nuechtern, Dominic Wichmann, Johannes Stamm, Tim Hardel, Holger Rohde, Martin Christner, Ann‐Kathrin Ozga, Stefan Steurer, Claudia Jafari, Hans Klose, Stefan Kluge, Marcel Simon, Marlene Fischer

**Affiliations:** ^1^ Department of Intensive Care Medicine University Medical Center Hamburg‐Eppendorf Hamburg Germany; ^2^ Department of Medical Microbiology, Virology and Hygiene University Medical Center Hamburg‐Eppendorf Hamburg Germany; ^3^ Center for Experimental Medicine, Institute of Medical Biometry and Epidemiology University Medical Center Hamburg‐Eppendorf Hamburg Germany; ^4^ Center for Diagnostics, Institute of Pathology With the Sections Molecular Pathology and Cytopathology University Medical Center Hamburg‐Eppendorf Hamburg Germany; ^5^ Department of Clinical Infectious Diseases Research Center Borstel Borstel Germany; ^6^ Department of Respiratory Medicine University Medical Center Hamburg‐Eppendorf Hamburg Germany

## Abstract

**Background:**

Obtaining non‐directed samples from the upper bronchial tree is easier to perform and poses fewer risks for critically ill patients than deep bronchoalveolar lavage (BAL). Since invasive pulmonary aspergillosis is associated with a high mortality in critically ill patients, timely diagnosis and rapid initiation of treatment are of utmost importance.

**Objectives:**

The objective of this study was to compare Galactomannan (GM) testing by Enzyme Immunoassay (EIA), GM Lateral Flow Assay (LFA) and the detection of *Aspergillus* DNA by Polymerase Chain Reaction (PCR) between directed BAL and non‐directed bronchial lavage (BL) in critically ill patients.

**Methods:**

In this prospective, exploratory pilot study, we analysed 120 samples from 40 patients admitted to 12 mixed intensive care units. Inclusion criteria required either risk factors for IPA or positive *Aspergillus* assessments and met the criteria published by the European Society of Clinical Microbiology and Infectious Diseases guidelines for IPA diagnosis. Both respiratory secretions and blood were collected. In each patient, LFA and PCR were performed on BAL, BL and blood serum, respectively. The EIA test was applied to the BL and BAL of each patient, and the serum of 24 patients. The study was registered on clinicaltrials.gov (NCT04848831).

**Results:**

In a total of 80 respiratory samples, *Aspergillus* GM EIA yielded optical density indices (ODI) ranging from 0.04 to ≥ 3.5. We observed a high correlation between BAL and BL samples for *Aspergillus* GM EIA (Pearson's *r* = 0.78 [95% CI 0.62, 0.88]; intraclass correlation coefficient 0.78). At an ODI cutoff of 0.8 for BAL and 1.2 for BL, the sensitivity of *Aspergillus* GM EIA was 0.94, while the specificity was 0.67. Increasing the BAL cutoff to 1.0 ODI improved the specificity to 0.86. *Aspergillus* PCR examination showed good agreement between the two compartments, with a Cohen's kappa coefficient of 0.75 (95% CI 0.48, 1.00). The correlation of *Aspergillus* GM LFA between BAL and BL was weak.

**Conclusions:**

Our findings demonstrate that the detection of *Aspergillus* GM using EIA or *Aspergillus* PCR in BL is comparable to that in BAL. Thus, BL samples can be reliably used for diagnosing invasive pulmonary aspergillosis.

**Abbreviations**
BAL: Bronchoalveolar Lavage
BL: Non‐Directed Bronchial Lavage
CI: Confidence Interval
COVID‐19: Coronavirus Disease 2019
EIA: Enzyme Immunoassay
GM: Galactomannan
ICC: Intraclass Correlation Coefficient
ICU: Intensive Care Unit
IPA: Invasive Pulmonary Aspergillosis
LFA: Lateral Flow Assay
PCR: Polymerase Chain Reaction
SARS‐CoV‐2: Severe Acute Respiratory Syndrome Coronavirus 2
TOST: Two One‐Sided Tests

## 1 Introduction

*Aspergillus* is a common saprophytic environmental fungus that can cause severe disease in humans through the inhalation or ingestion of its airborne conidia [1, 2]. Invasive pulmonary aspergillosis (IPA) is a severe disease with a high mortality rate up to 92% [3, 4]. Traditionally, IPA has been accepted to occur in immunocompromised patients with specific risk factors predisposing for fungal infection [1, 2]. However, in the last two decades, IPA has been affecting more and more critically ill patients without traditional risk factors [4, 5]. Additionally, IPA has been observed more frequently during influenza outbreaks since 2009 [6] and the SARS‐CoV‐2 pandemic since 2020 [7, 8].

The diagnosis of IPA remains a major challenge [2]. Although numerous biochemical, immunological, molecular, microbiological and cytological methods of detection are available, histological evidence is the only way to diagnose proven IPA [9]. Of note, obtaining a biopsy in critically ill patients is not always possible and frequently complicated by coagulopathy or escalated invasive ventilation [10, 11]. In recent years, the *Aspergillus* galactomannan (GM) enzyme immunoassay (EIA) in bronchoalveolar lavage (BAL) fluid has become the preferred method for the diagnosis of IPA in critically ill patients [9, 12]. Yet, the interpretation of GM EIA and thus, IPA diagnosis is not uniform and consistent [3, 13, 14, 15]. In addition, microbiological work‐up includes the lateral flow assay (LFA) and the polymerase chain reaction (PCR). The LFA is a point‐of‐care test for the detection of cellular wall components that is also feasible at the bed‐side and in small laboratories [16, 17] Of note, the ability of *Aspergillus* PCR to differentiate between colonisation and invasive disease is limited [18].

During the SARS‐CoV‐2 pandemic, concerns regarding airborne transmission led to a decline in bronchoscopic assessment resulting in a particular challenge in diagnosing IPA [19]. Thus, secretions obtained from the lower airways via non‐directed bronchial lavage (BL) were analysed to diagnose COVID‐19‐associated pulmonary aspergillosis (CAPA) [7, 20, 21, 22, 23].

**FIGURE 1:**
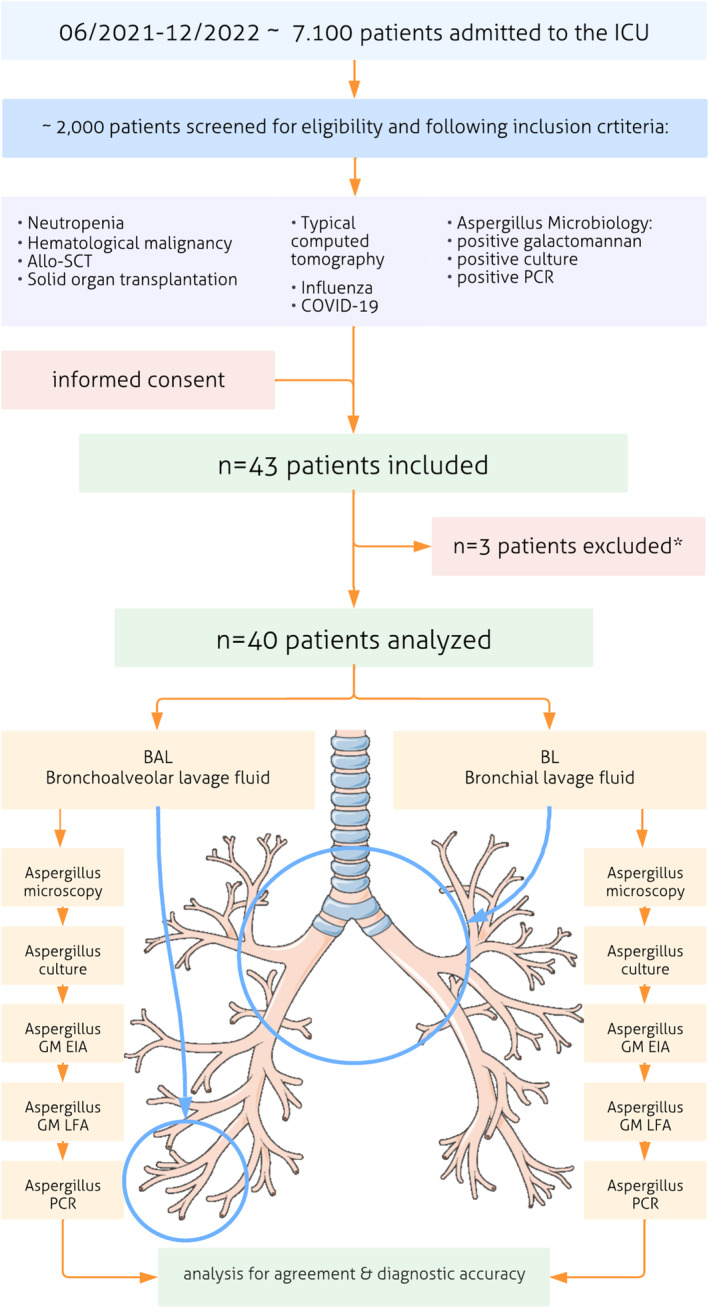
Flow of participants. Allo‐SCT, allogeneic stem cell transplantation, COVID‐19: Coronavirus disease 2019, EIA, enzyme immunoassay, GM, galactomannan, LFA, lateral flow assay, PCR, polymerase chain reaction. *Patients excluded, bronchoscopy not performed (*n* = 2), sample not tested (*n* = 1).

Bronchoalveolar lavage and BL are distinct diagnostic procedures targeting different regions of the respiratory tract (Figure 1). While BAL samples the distal airways and alveoli using a larger volume of saline (100–200 mL), BL focuses on the proximal bronchial tree with a smaller saline volume (< 50 mL), making it more rapid and less invasive to perform. Bronchial lavage is particularly suitable for critically ill, non‐ventilated patients due to its minimal impact on gas exchange.

The aim of this pilot study was to compare quantitative and qualitative test methods (*Aspergillus* GM EIA and LFA, PCR for *Aspergillus* DNA) in different respiratory secretions from critically ill patients. In addition, we sought to obtain information for sample size calculation for a further larger prospective study.

## 2 Material and Methods

### 2.1 Study Design and Participants

This single‐center, prospective, exploratory pilot study included critically ill patients admitted to the Department of Intensive Care Medicine at the University Medical Center Hamburg‐Eppendorf between June 24, 2021, and December 8, 2022. Figure 1 shows inclusion criteria and presents the flow of participants. Patients were included, if they had risk factors for IPA or microbiological or radiological assessment indicative for *Aspergillus* spp. according to the criteria published by the European Society of Clinical Microbiology and Infectious Diseases guidelines for IPA diagnosis [15]. All ICU patients (both medical and surgical) were screened 2 to 3 times per week based on one or more of the following inclusion criteria: neutropenia, hematologic malignancy, allogeneic‐stem cell transplantation, solid organ transplantation, influenza, COVID‐19, *Aspergillus*‐type computed tomography signs (halo sign, air crescent sign), or *Aspergillus*‐positive microbiologic tests (GM EIA, culture, PCR). Patients were excluded if they were unconscious without a legal guardian, had contraindications to bronchoscopy, or had no indication for the procedure. A total of 2000 patients were screened and after written informed consent, 43 were included in the study. Three patients were excluded from the analysis (bronchoscopy not performed: *n* = 2; sample not tested: *n* = 1).

Figure 1 shows inclusion criteria for study participants as well as an overview of the diagnostic procedures performed in two different compartments of the lung: non‐directed bronchial lavage (BL) in the upper bronchial tree and bronchoalveolar lavage (BAL) in the lower bronchial treee.

### 2.2 Ethical Approval

Because of the prospective study design, an ethical approval was required. The study protocol was reviewed and approved by the Ethics Committee of the Hamburg Chamber of Physicians (protocol no. 2021‐10487‐BO‐ff) on 3 March 2021. Written informed consent was obtained from patients or their legal representatives. The study was registered under the number NCT04848831 on clinicaltrials.gov.

### 2.3 Setting

The University Medical Center Hamburg‐Eppendorf is a quaternary care hospital that is located in Northern Germany and offers advanced specialised care and complex procedures. The Department of Intensive Care Medicine comprises 12 multidisciplinary intensive care units (ICUs) with a total capacity of up to 140 ICU beds. The department provides intensive care for all adult critically ill patients admitted to the hospital covering medical and surgical units.

### 2.4 Bronchoscopy

Bronchoscopy with BL and BAL was performed by pulmonologists or intensivists experienced in the bronchoscopy of critically ill patients. For BL, 20 mL of sterile isotonic saline were instilled into the central airway through the working channel of the bronchoscope. The saline solution was then reaspirated, ensuring that at least 5 mL were preserved for further examination. This procedure was performed in analogy to a non‐bronchoscopic BL [24, 25]. For BAL, sterile isotonic saline was instilled in aliquots of 20 mL from a wedged position of the bronchoscope into the appropriate area of the lung determined from radiographic images. The fluid was then reaspirated. The first portion of reaspirated fluid was discarded because it consists of saline solution with little bronchial fluid while the subsequent portions were pooled and preserved for further analysis [26].

### 2.5 Microbiological Assays

For *Aspergillus* culture, samples were centrifuged at 3000 *g* for 5–10 min, and the resulting pellet was resuspended in 1 mL supernatant. To prevent bacterial contamination, Sabouraud dextrose‐agar with gentamicin and chloramphenicol was inoculated with 50–100 μL of the suspension and incubated at 30°C for 14 days thereafter. Culture isolates were identified by matrix‐assisted laser desorption ionisation time‐of‐flight mass spectrometry fingerprinting or internal transcribed spacer sequencing.

*Aspergillus* GM was detected in BL fluid, BAL fluid, and serum samples using the Platelia *Aspergillus* EIA, Bio‐Rad.

The IMMY sōna *Aspergillus* GM LFA was used after pre‐treatment with EDTA buffer and heating to 120°C for 5–8 min. The test lines' intensities were read after 30 min using an automated cube reader (sōna LFA Cube Reader, IMMY, Norman, OK, USA) and displayed in ODI [16].

PCR amplification was carried out using specific primers targeting *Aspergillus* DNA sequences. The PCR reaction mixture contained the extracted DNA, primers, nucleotides and a DNA polymerase enzyme. The reaction involved cycles of heating and cooling, which allowed the primers to bind to the target DNA and the DNA polymerase to copy and amplify the target sequences. The resulting PCR products were analysed using gel electrophoresis or real‐time PCR to determine the presence of *Aspergillus* DNA.

Histopathological examination of BAL fluid was performed after haematoxylin–eosin and Grocott methenamine silver staining to identify *Aspergillus* hyphae, typical filaments and mycelium. These stains help in visualising fungal structures, aiding in accurate diagnosis.

### 2.6 Data Collection

Data was collected using an electronic patient data management system (PDMS, Integrated Care Manager V10 and ICMiq V1.3, Drägerwerk, Lübeck, Germany). This system recorded comprehensive patient information, including age, sex, admission diagnosis, comorbidities, risk factors for invasive pulmonary aspergillosis (IPA), antifungal treatment, details on ICU management and survival status at 30 and 90 days. Microbiological assessments were thoroughly documented, including the identification of *Aspergillus* spp., the source of sampling (BL or BAL), and results from *Aspergillus* GM EIA, LFA, and PCR tests. Additionally, *Aspergillus* GM EIA, LFA, and ß‐D‐Glucan levels in serum were recorded, along with results from microscopic examinations of BL and BAL fluids. Details on macroscopic bronchoscopy findings, and chest X‐ray or computed tomography results, were also documented.

### 2.7 Statistical Analysis

We used descriptive statistics to characterise the study population. For categorical variables, absolute and relative frequencies are given. For continuous variables, we report the median with interquartile range (IQR).

Pearson (for normally distributed data) or spearman correlation coefficients (for not normally distributed data) and the intraclass correlation coefficient (ICC, based on normally distributed data obtained via log‐transformation) were used to analyse the correlation and agreement between sample sources. For skewed data, log‐transformation was used to obtain normal distributed data. Cohen's kappa coefficient was used to determine the agreement of PCR results between upper and lower bronchial compartments for binary outcome.

Sensitivity and specificity were calculated for the *Aspergillus* GM EIA using specific cut‐off values for BAL (0.8 ODI) in accordance with the proposed definition to probable IPA in the critical care setting, as outlined by the EORTC/MSGERC (European Organisation for Research and Treatment of Cancer/Mycoses Study Group Education and Research Consortium) Definitions 2021 [9]. Furthermore, sensitivity and specificity were calculated for the *Aspergillus* GM EIA using specific cut‐off values for BAL (1.0 ODI) in accordance with the 2024 consensus definitions from ESGCIP, EFISG, ESICM, ECMM, MSGERC, ISAC, and ISHAM [15]. IPA Sensitivity and specificity values were subsequently determined based on these pre‐defined cut‐off thresholds.

Statistical analyses were performed using SPSS (IBM SPSS Statistics for Windows, Version 27.0. Armonk, NY, USA) and R version 4.1.2 with packages *blandr, psych, cutpointr, TOSTER*.

### 2.8 Artificial Intelligence (AI) Tools

This manuscript was drafted with the assistance of DeepL and ChatGPT as language tools for translation and correction of scientific texts. DeepL is an AI‐based translation tool, and ChatGPT is a language model trained on a large corpus of text. Both tools were used to improve the accuracy and clarity of the language used in the manuscript.

## 3 Results

### 3.1 Study Population

Between June 2021 and December 2022, we enrolled 40 consecutive patients who were either at risk of IPA or had *Aspergillus* spp. detected in respiratory secretions (Figure 1). The median age of the participants was 64 years (ranging from 52 to 69 years). The majority of patients included in the study were admitted to the ICU due to respiratory failure (*n* = 19, 47.5%) or infectious diseases resulting from haematological malignancy (*n* = 8, 20%). Of the 40 study participants, 21 (53%) required invasive mechanical ventilation, and two (5%) required extracorporeal membrane oxygenation. The study also included patients with severe influenza pneumonia (*n* = 2, 5%), and those treated for COVID‐19 (*n* = 5, 12.5%). The 30‐day mortality rate after ICU admission was 12.5%, the 90‐day mortality was 30% (Table 1).

**TABLE 1:** Baseline characteristics and clinical outcomes of the study population.

Demographics	Median (IQR) or *n* (%)
Sex (female)	15 (37.5)
Age (years)	64 (52–69)
Reason for ICU admission	
Respiratory failure	19 (47.5)
Sepsis due to haematological malignancy	8 (20)
Surgical	6 (15)
Neurological	5 (12.5)
Sepsis following solid organ transplantation	2 (5)
Risk factors for IPA	
Neutropenia	2 (5)
Haematological malignancy	10 (25)
Allogeneic stem cell transplantation	5 (12.5)
Solid organ transplantation	3 (7.5)
COVID‐19	5 (12.5)
Influenza	2 (5)
ICU stay	
Ventilation	
No mechanical ventilation	11 (27.5)
NIV	8 (20)
Invasive mechanical ventilation	21 (52.5)
ECMO support	2 (5)
Antifungal treatment	28 (70)
Azole (voriconazole or isavuconazole)	23 (57.5)
Echinocandin (caspofungin or anidulafungin)	3 (7.5)
Liposomal amphotericin B	13 (32.5)
Antifungal prophylaxis	7 (17.5)
Co‐infections	
Respiratory bacterial infections	23 (57.5)
Other bacterial infections	26 (65)
Respiratory viral infections	18 (45)
Other viral infections	8 (20)
Other fungal infection	13 (32.5)
*Candida* spp.	12 (30)
*Trichosporon asahii*	1 (2.5)
Outcome	
30‐day mortality	5 (12.5)
90‐day mortality	12 (30)

*Note:* Baseline demographic data, reason for ICU admission, ICU management details, and outcomes were collected from 40 patients who underwent bronchoscopy with non‐directed bronchial lavage (BL) and bronchoalveolar lavage (BAL) to investigate for aspergillosis. Continuous variables are presented as median (1st to 3rd) quartile, and categorical variables are presented as *n* (%).

Abbreviations: COVID‐19, Coronavirus disease 2019; ECMO, extracorporeal membrane oxygenation; ICU, intensive care unit; IPA, invasive pulmonary aspergillosis; NIV, non‐invasive ventilation.

The detailed factors predisposing for IPA and previous *Aspergillus* detection are listed in Table S1. Nineteen (47.5%) of the patients met more than one inclusion criterion. In three patients (7.5%), *Aspergillus fumigatus* growth was detected in both BAL and BL. Additionally, samples from another three patients showed cultural growth of *Aspergillus* spp. in only one of the two compartments examined. GM EIA determined optical density indices (ODI) ranged from 0.04 to ≥ 3.5 (Table S1). *Aspergillus* DNA was detected in five BAL (12.5%) and in seven BL (17.5%) samples, respectively (Table S1).

**FIGURE 2:**
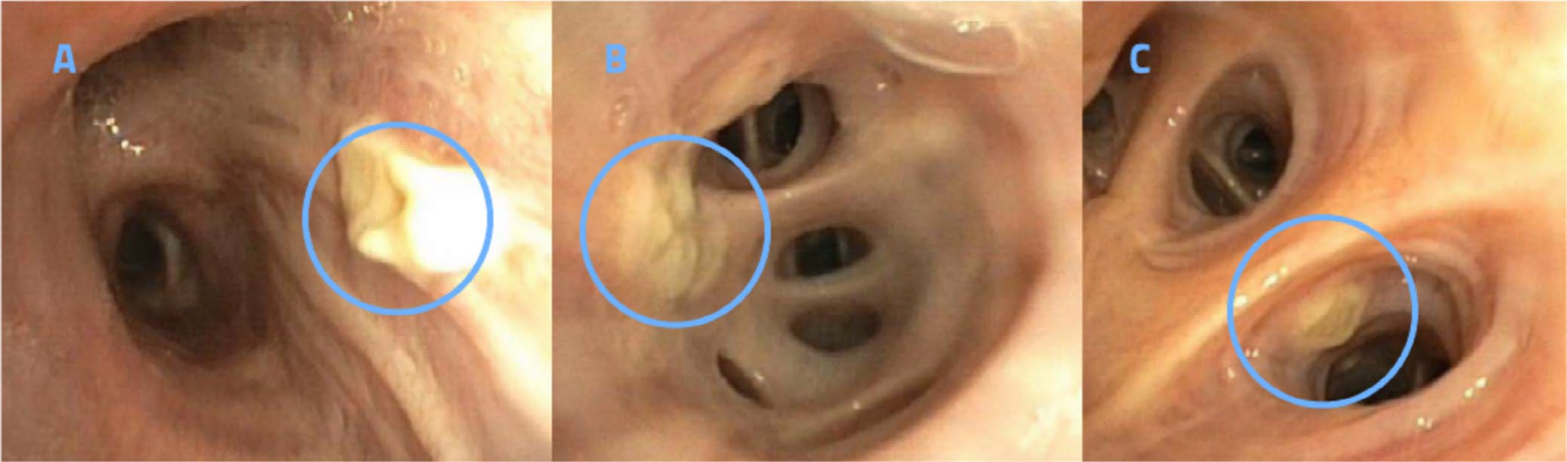
Bronchoscopic images of bronchial aspergillosis (A) the right main bronchus, (B) the left basal lower lobe bronchus, and (C) the lingular bronchus. The patient had been previously treated for COVID‐19, with an initial positive result for *Aspergillus* galactomannan, without cultural evidence of *Aspergillus* spp. having been obtained at that time.

Bronchoscopy revealed visible lesions indicative of tracheobronchial aspergillosis in six patients. An example is shown in Figure 2, illustrating the characteristic appearance observed during the procedure.

### 3.2 Correlation and Agreement Between Upper and Lower Bronchial Tree

**FIGURE 3:**
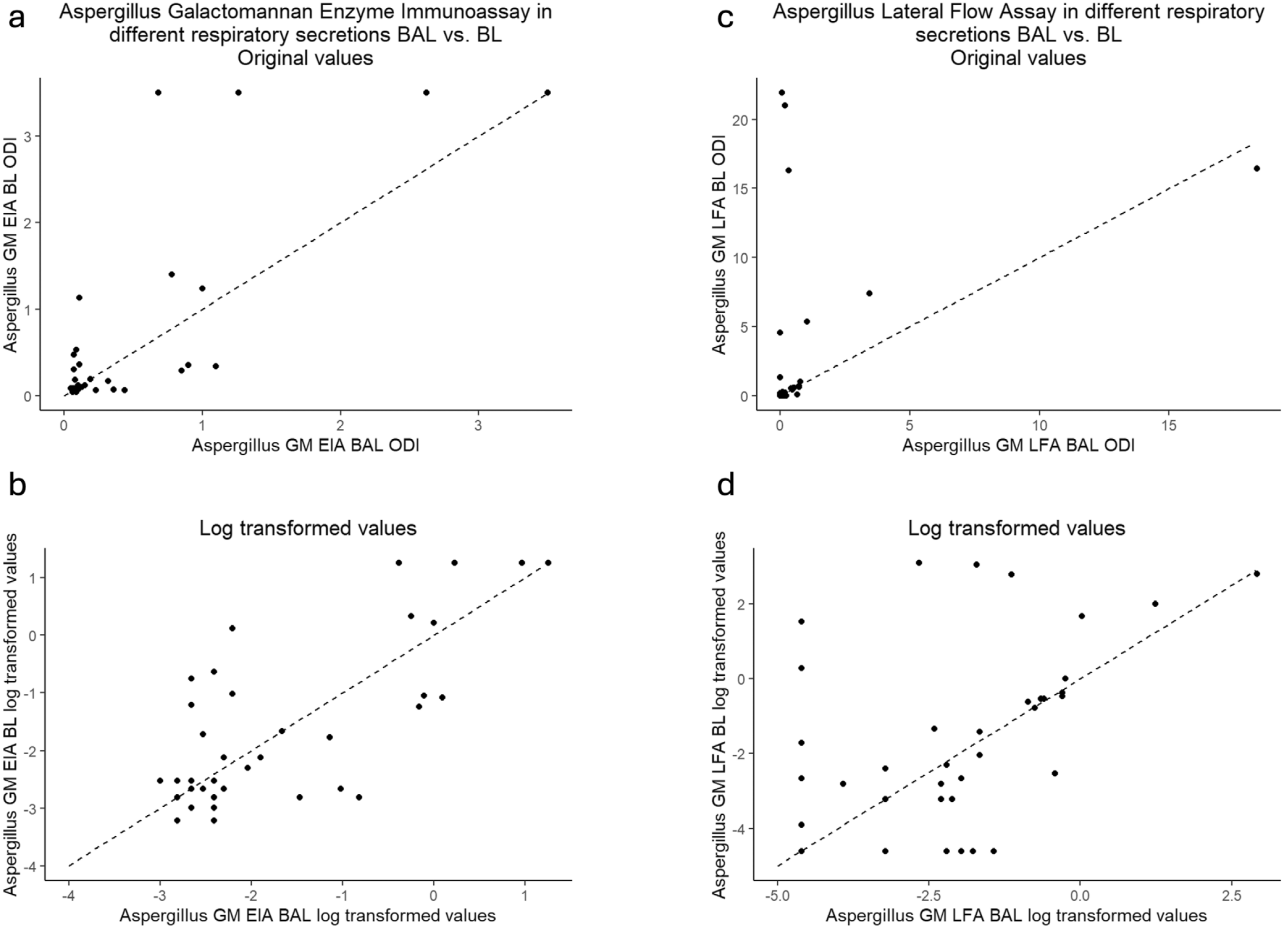
(A–D) Scatterplots illustrating the comparison of *Aspergillus* galactomannan (GM) detection methods in bronchoalveolar lavage (BAL) and bronchial lavage (BL) samples. The first figure shows the galactomannan lateral flow assay (GM LFA), while the second figure represents the galactomannan enzyme immunoassay (GM EIA). For each method, the top plots display the original optical density index (ODI) values, and the bottom plots present log‐transformed values to better visualise the data distribution and linear relationships. Dashed diagonal lines in all plots indicate the line of equality (*y* = *x*), representing perfect agreement between BAL and BL results. These comparisons highlight differences in performance and agreement between the two diagnostic methods across sample types.

Quantitative comparison of *Aspergillus* GM EIA in the examined secretions showed high correlation and agreement, with a Pearson correlation coefficient of 0.78 (95% CI 0.62, 0.88) for log‐transformed data and an ICC of 0.78. The comparison of *Aspergillus* GM LFA showed a moderate positive correlation or agreement, with a Pearson correlation coefficient of 0.50 (95% CI 0.22, 0.70) and an ICC of 0.47 (Table 2). Table 2 shows the Spearman correlation based on the original (skewed) data. Correlations of EIA and LFA between the two compartments are shown in Figure 3A–D. For *Aspergillus* PCR examination, we observed good agreement between the two compartments, with a Cohen's kappa coefficient of 0.75 (95% CI 0.48, 1.01; Table 2).

**TABLE 2:** Coefficients of correlation and agreement of *Aspergillus* EIA, LFA, and PCR between the upper and lower bronchial tree.

Data	Pearson (95% CI)	Spearman	ICC	Kappa coefficient (95% CI)
*Aspergillus* GM EIA				
Original		0.63	0.82	
Log‐transformed	0.78 (0.62, 0.88)		0.78	
*Aspergillus* GM LFA				
Original		0.49	0.29	
Log‐transformed	0.50 (0.22, 0.70)		0.47	
*Aspergillus* PCR				0.75 (0.48, 1.01)

Abbreviations: CI, confidence intervall; EIA, enzyme immunoassay; GM, galactomannan; ICC, interclass correlation coefficient; LFA, lateral flow assay; PCR, polymerase chain reaction.

### 3.3 Sensitivity and Specificity

The sensitivity of the *Aspergillus* GM EIA was 0.94 with a cutoff of 0.8 ODI for BAL samples and 1.2 ODI for BL samples, while the specificity was 0.67. When the cut‐off was adjusted to 1.0 ODI for true positive BAL samples, the specificity increased to 0.86 (Table 3). Specificities and sensitivities were not determined due to insufficient agreement with the LFA values.

**TABLE 3:** Sensitivity and specificity of *Aspergillus* galactomannan enzyme‐linked immunosorbent assay for upper and lower bronchial tree samples using different cut‐off values.

GM EIA cut‐off for BL	GM EIA cut‐off for BAL			
	0.8 ODI		1.0 ODI	
	Specificity	Sensitivity	Specificity	Sensitivity
0.8 ODI	0.67	0.90	0.86	0.91
1.0 ODI	0.67	0.90	0.86	0.91
1.2 ODI	0.67	0.94	0.86	0.94
2.0 ODI	0.56	0.97	0.71	0.97
> 3.5 ODI	0.56	0.97	0.71	0.97

*Note:* The sensitivity and specificity of the *Aspergillus* GM EIA for bronchial lavage (BL) and bronchoalveolar lavage (BAL) samples with various cut‐off values. The values in the table represent the proportion of true negative (specificity) and true positive (sensitivity) results obtained for each respective cut‐off value.

Abbreviations: EIA, enzyme immunoassay; GM, galactomannan; ODI, optical density index.

## 4 Discussion

The main findings of our study are: First, *Aspergillus* GM EIA showed a high level of agreement between BAL and BL samples from critically ill patients. Second, the qualitative *Aspergillus* GM EIA results from BAL and BL showed excellent sensitivity and specificity. Both findings suggest that non‐directed BL is a reliable alternative to BAL in critically ill patients.

Non‐directed BL has previously been reported to represent a safe and cost‐effective procedure for critically ill patients [27]. It can be easily performed by a trained member of ICU staff without the need for bronchoscopy [27]. In the present study, non‐directed, bronchoscopic BL was compared to directed BAL. This comparison is of clinical relevance in the context of critically ill patients, as it considers not only the duration of the procedures, with BL being substantially more time‐efficient, but also their impact on gas exchange. A rapid diagnostic procedure is particularly important for patients with mild or moderate respiratory failure, who may require oxygen therapy, high‐flow nasal oxygen, or non‐invasive ventilation to avoid intubation, mechanical ventilation, and sedation. Therefore, this vulnerable patient population may benefit most from avoiding any additional respiratory distress, such as the application of large amounts of saline to the lower airways.

Regarding the quality of microbiological examinations, a prospective study showed an accuracy of BL in diagnosing ventilator‐associated pneumonia, with a sensitivity of 50% and a specificity of 95% when compared to post‐mortem histological confirmation [28]. Another clinical service evaluation in 39 critically ill patients demonstrated that elective microbiologic surveillance by BL can be useful in guiding antibiotic therapy, both by initiating targeted therapy at the time of diagnosis and by making early decisions about de‐escalation of antibiotic therapy [24].

A recent proof‐of‐concept study involving 32 intubated patients, demonstrated that the *Aspergillus* GM EIA for the detection of IPA using deep BL yielded comparable results to the *Aspergillus* GM EIA in BAL fluid. However, the authors acknowledged that the study's limitation was the clinical selection of patients with a high pretest probability of IPA, resulting in highly positive GM results (mean ODI > 3). To further assess the clinical utility of *Aspergillus* GM EIA in BL, the authors suggested to consider lower ODI values to establish a reliable cut‐off [29]. By including a heterogenous study population with a lower pretest probability compared with the aforementioned study of Rothe et al., our study design closed the gap. The results of our study demonstrated good agreement, even with ODI values below 0.5 for *Aspergillus* GM EIA.

The combination of BL and a point‐of‐care test, such as LFA, would be a valuable diagnostic tool that could also be used beyond highly specialised intensive care facilities. The accuracy of the *Aspergillus*‐specific lateral flow device (LFD) assay and the sōna *Aspergillus* GM LFA have been evaluated for the diagnosis of IPA in patients with hematologic malignancies, showing promising results in BAL [30]. In 178 critically ill patients, the *Aspergillus* GM LFA demonstrated a sensitivity of 0.94 and a specificity of 0.81 for the diagnosis of IPA in BAL [31]. Importantly, this study did not assess the diagnosis of IPA from BL limiting the comparability with our findings. Autier and colleagues assessed the diagnostic performance of LFA in 238 patients with CAPA. Interestingly, the LFA from non‐directed BL showed a sensitivity of 80% and a specificity of 83% for the detection of IPA when using the 1.0 OD cut‐off [32]. Similarly, another diagnostic accuracy study in patients with CAPA found an agreement of 93% for LFA from tracheal aspirates [23]. While the *Aspergillus* GM LFA has shown promising test performance in patients with hematologic malignancies and CAPA, its role for the diagnosis of IPA in a heterogenous spectrum of ICU patients remains to be determined [13]. The heterogeneity of our study population, including a broad range of medical and surgical patients, might account for the moderate agreement of GM LFA between BAL and BL.

The detection of *Aspergillus* DNA from PCR is characterised by a low sensitivity, which might be attributable to methodological variance [18]. Likewise, we found positive PCR in only a small subset of patients (Table S1). However, the agreement of *Aspergillus* PCR between the lower and the upper bronchial compartments the correlation was good, with a kappa coefficient of 0.75 (95% CI: 0.48–1.01).

In addition to being a procedure for collecting lavage fluid for analysis, bronchoscopy is able to visualise macroscopic lesions of invasive tracheobronchial aspergillosis, a subtype of IPA [33, 34]. In our study, lesions of tracheobronchial aspergillosis were bronchoscopically visible in 6 (15%) of the patients (Table S1 and Figure 2). Therefore, bronchoscopy by a trained examiner remains an important diagnostic tool for patients with suspected IPA, in order to allow for visualisation of mucosal lesions and structural alterations.

### 4.1 Limitations

One limitation of our study is the small sample size due to the exploratory nature of the study design. It is possible that a larger confirmatory approach might identify agreement between *Aspergillus* GM LFA in BAL and BL samples.

Additionally, the study population was limited to patients at risk of IPA or with microbiological detection of *Aspergillus* spp. in respiratory secretions, which may limit the generalizability of our findings to other patient populations. Furthermore, the single‐center study was conducted at a quaternary care hospital, further limiting the external validity of our results to other settings.

## 5 Conclusions

In the present study, we found a high agreement of *Aspergillus* GM EIA between BAL and BL. Thus, secretions obtained by non‐directed BL from the upper bronchial tree may be used equally to directed BAL in the detection of IPA. The role of BL for the diagnosis of IPA in critically ill patients should be strengthened, as it is easier to perform and less invasive than BAL. In addition, the novel findings of the present study allow for a sample size estimation to design future confirmatory studies. Future studies might focus on identifying the optimal cut‐off value for *Aspergillus* GM EIA in BL in a large and heterogenous population of critically ill patients.

## Author Contributions

**Maria Schroeder:** conceptualization, investigation, funding acquisition, writing – original draft, visualization, validation, methodology, project administration, data curation, writing – review and editing, formal analysis, supervision. **Mohamad Abd Raboh:** investigation, formal analysis, data curation. **Annika Nuechtern:** investigation, formal analysis, data curation. **Dominic Wichmann:** writing – review and editing, conceptualization. **Johannes Stamm:** investigation. **Tim Hardel:** investigation. **Holger Rohde:** resources, conceptualization. **Martin Christner:** resources, conceptualization. **Ann‐Kathrin Ozga:** software, formal analysis. **Stefan Steurer:** investigation. **Claudia Jafari:** writing – review and editing. **Hans Klose:** investigation. **Stefan Kluge:** conceptualization, writing – review and editing, supervision. **Marcel Simon:** conceptualization, investigation, writing – review and editing. **Marlene Fischer:** conceptualization, investigation, writing – original draft, validation, methodology, writing – review and editing, supervision.

## Consent

All authors agreed to the publication of the current version of the article.

## Supporting information


Table S1.

## Data Availability

The datasets used and/or analysed during the current study are available from the corresponding author on reasonable request.
