# Supplementary material for: Comparison of the Equivalence of Aspergillus Antigen and PCR Results Between Non‐Directed Bronchial Lavage and Bronchoalveolar Lavage—A Prospective Exploratory Pilot Study in Critically Ill Patients
Source: Mycoses. 2025 Feb 3;68(2):e70029. doi: 10.1111/myc.70029 (PMC11790511; doi:10.1111/myc.70029)
Supplement: Supplementary file 1 — Table S1. [file MYC-68-e70029-s001.docx]

| Inclusion criteria | Aspergillus culture | | Galactomannan EIA | | | Galactomannan LFA | | | Aspergillus PCR | | Microscopy | | ß-D-Glucan | Tracheobronchial aspergillosis | BAL fluid |
| --- | --- | --- | --- | --- | --- | --- | --- | --- | --- | --- | --- | --- | --- | --- | --- |
|  | BAL | BL | BAL | BL | Serum | BAL | BL | Serum | BAL | BL | BAL | BL | Serum | Bronchoscopy | Instilled/Recovered [ml] |
| Macroscopic lesions of tracheobronchial aspergillosis |  |  | 0.05 | 0.08 |  | 0.53 | 0.48 | 0.56 | negative | negative |  |  | 46.8 | positive | 120/80 |
| SOT, TCT: nodular infiltrates | A. fumigatus | A. fumigatus | >3.5 | >3.5 |  | 0.22 | 16.2 | 0.51 | positive | positive |  | Mycelium | 92.5 | positive | 160/70 |
| SARS-CoV2, TCT: ground-glass opacity, BAL: Aspergillus GM + |  |  | 0.09 | 0.53 |  | 0.07 | 0.06 | 0.24 | negative | negative |  |  | 3.8 | positive | 100/80 |
| SARS-CoV2, BAL: Aspergillus GM + |  |  | 1.26 | >3.5 | >3.5 | 0.11 | 20.9 | 1.2 | positive | positive |  | Mycelium |  | positive | 140/90 |
| BAL: Aspergillus GM + |  |  | 0.44 | 0.06 | 0.27 | 0.14 | 0 | 0.11 | negative | negative |  |  | 20.8 | positive | 100/51 |
| TCT: ground-glass opacity, halo sign |  |  | 0.10 | 0.07 | 0.12 | 0 | 0 | 0.08 | negative | negative |  |  | 276.1 | positive | 100/50 |
| Hematological disease, BAL: Aspergillus culture + |  |  | 0.09 | 0.04 |  | 0.51 | 0.57 | 0.47 | negative | negative |  |  | 3.5 |  | 160/80 |
| Neutropenia, SCT, TCT: ground-glass opacity, BAL: Aspergillus culture + |  |  | 1.00 | 1.24 |  | 0.24 | 0.68 | 0.54 | negative | negative |  |  | 3.5 |  | 100/50 |
| SCT, TCT: ground-glass opacity, BAL: Aspergillus GM + |  |  | 0.07 | 0.30 |  | 0.66 | 0.55 | 0.79 | negative | negative |  |  | 3.5 |  | 140/70 |
| TCT: ground-glass opacity |  |  | 0.06 | 0.04 |  | 0.70 | 0.91 | 0.62 | negative | negative |  |  | 3.2 |  | 120/95 |
| BAL: Aspergillus culture + |  |  | 0.11 | 0.36 |  | 0.43 | 0.42 | 0 | negative |  |  |  | 6.4 |  | 120/85 |
| SARS-CoV2, TCT: ground-glass opacity |  |  | 0.06 | 0.06 |  | 0.39 | 0.49 | 0.47 | negative | negative |  |  | 3.7 |  | 120/70 |
| Hematological disease, SCT TCT: ground-glass opacity, BAL: Aspergillus GM + |  |  | 0.23 | 0.06 |  | 0.06 | 0.10 | 0.11 | negative | negative |  |  | 4.2 |  | 180/91 |
| TCT: new cavity |  |  | 0.07 | 0.47 |  | 0.11 | 0 | 0.21 | negative | negative |  |  | 3.2 |  | 160/51 |
| TCT: nodular infiltrates, BAL: Aspergillus culture + |  |  | 2.62 | >3.5 |  | 0.96 | 5.31 | 0.29 | positive | positive |  |  | 8.9 |  | 120/58 |
| TCT: nodular infiltrates |  |  | 0.07 | 0.05 | 0.05 | 0.07 | 0.04 | 0.15 | negative | negative |  |  | 20.9 |  | 140/80 |
| BAL: Aspergillus culture + |  |  | 0.13 | 0.10 | 0.13 | 0 | 0 | 0.11 | negative | negative |  |  | 0.13 |  | 120/68 |
| SOT, BAL: Aspergillus culture + |  |  | 0.09 | 0.05 |  | 0 | 0.02 | 0 | negative | negative |  |  | 13.2 |  | 180/90 |
| SCT |  | A. fumigatus | 0.68 | >3.5 | 1.12 | 0.02 | 21.1 | 0.05 | positive | positive |  | Mycelium | 1.12 |  | 120/70 |
| Hematological disease, BAL: Aspergillus GM + |  |  | 0.78 | 1.40 |  | 0.05 | 0.01 | 0 | negative | negative |  |  | 5.8 |  | 100/71 |
| SARS-CoV2, BAL: Aspergillus GM + |  |  | 0.07 | 0.08 | 0.01 | 0 | 0.02 | 0.17 | negative | negative |  |  | 0.04 |  | 120/77 |
| BAL: Aspergillus culture + |  |  | 0.11 | 1.13 | 0.08 | 0.07 | 0.19 | 0. | negative | positive |  |  |  |  | 140/105 |
| BAL: Aspergillus GM + |  |  | 0.07 | 0.07 | 0.23 | 0.01 | 0.01 | 0 | negative | negative |  |  | 2.9 |  | 100/38 |
| BAL: Aspergillus GM + |  |  | 0.07 | 0.07 | 0.05 | 0.01 | 0.02 | 0.08 | negative | negative |  |  | 2.9 |  | 115/42 |
| BAL: Aspergillus GM + |  |  | 0.09 | 0.06 |  | 0.36 | 0.01 | 0.11 | negative |  |  |  | 4.8 |  | 80/40 |
| Hematological disease, TCT: ground-glass opacity |  |  | 0.15 | 0.12 |  | 0.06 | 0 | 0.08 | negative | negative |  |  |  |  | 60/35 |
| TCT: ground-glass opacity, halo sign |  |  | 0.32 | 0.17 | 0.05 | 0 | 1.08 | 0.23 | negative | negative |  |  | 7.5 |  | 100/61 |
| Neutropenia, SCT |  |  | 0.08 | 0.24 | 0.04 | 0 | 0 | 0 | negative | negative | Mycelium | Mycelium | 121 |  | 60/40 |
| Hematological disease |  |  | 1.10 | 0.34 | 0.06 | 0.62 | 0.43 | 0.03 | negative | negative |  |  | 11.4 |  | 80/35 |
| SOT, SARS-CoV2, TCT: ground-glass opacity |  |  | 0.85 | 0.29 | 0.08 | 0 | 0.10 | 0.1 | negative | negative |  |  | 3.8 |  | 80/35 |
| Hematological disease |  |  | 0.19 | 0.19 | 0.08 | 0 | 0 | 0 | negative | negative |  |  | 3.5 |  | 120/55 |
| TCT: ground-glass opacity, BAL: Aspergillus GM + | A. fumigatus | A. fumigatus | >3.5 | >3.5 | 0.61 | 18.1 | 16.4 | 0.17 | positive | positive | Mycelium | Mycelium | 8.4 |  | 100/41 |
| BAL: Aspergillus GM + |  | A. fumigatus | 0.90 | 0.35 | 0.05 | 0 | 4.50 | 1.2 | negative | positive |  |  | 3 |  | 60/23 |
| Hematological disease |  |  | 0.10 | 0.12 | 0.12 | 0 | 0.14 | 0 | negative | negative |  |  |  |  | 100/55 |
| BAL: Aspergillus GM + | A. fumigatus |  | 0.36 | 0.07 | 0.07 | 0.15 | 0.00 | 0.01 | negative | negative |  |  | 9.5 |  | 100/45 |
| TCT: ground-glass opacity, BAL: Aspergillus GM + |  |  | 0.06 | 0.08 | 0.04 | 0 | 0 | 0 | negative | negative |  |  | 3 |  | 120/72 |
| BAL: Aspergillus culture + |  |  | 0.09 | 0.08 | 0.05 | 0.01 | 0 | 0.06 | negative | negative |  |  | 3 |  | 120/40 |
| BAL: Aspergillus culture + |  |  | 0.08 | 0.07 | 0.05 | 0.10 | 0 | 0 | negative | negative |  |  | 3 |  | 120/55 |
| Influenza, TCT: ground-glass opacity, BAL: Aspergillus GM + |  |  | 0.09 | 0.06 | 0.04 | 0.03 | 0.02 | 0 | negative | negative |  |  | 12.5 |  | 100/35 |
| Influenza | A. fumigatus | A. fumigatus | >3.5 | >3.5 | 0.11 | 3.43 | 7.4 | 0.03 | positive | positive | Mycelium | Mycelium | 7.5 |  | 100(35) |

**Supplemental Table 1.** Inclusion criteria and mycological work up

This table shows all patients (n=40) with results from bronchoalveolar lavage (BAL) and bronchial lavage (BL), presented in an anonymized form. Allo-SCT: Allogeneic Stem Cell Transplantation, COVID-19: Coronavirus Disease 2019, EIA: Enzyme Immunoassay, GM: Galactomannan, LFA: Lateral Flow Assay, PCR: Polymerase Chain Reaction, TCT: Thoracic computed tomography
